# Supplementary material for: Are carbon emissions trading and green financial instruments synergistic? -Comprehensive quantitative research based on content analysis
Source: PLoS One. 2024 Mar 7;19(3):e0298601. doi: 10.1371/journal.pone.0298601 (PMC10919601; doi:10.1371/journal.pone.0298601)
Supplement: S1 Data — (ZIP) [file pone.0298601.s001.zip › Data/地级市Z_F工作报告.zip!/╦─┤¿/π≥╓▌╩╨/└┤╫╘╣1⁄2╓┌║┼í╛┬φ┐╦╩2╛▌═°í┐_2018.docx]

各位代表：

现在，我代表市人民政府，向大会报告工作，请予审查，并请市政协委员提出意见。

2017年工作回顾

2017年，在省委、省政府和市委的正确领导下，在市人大、市政协的监督支持下，我们全力以赴稳增长，攻坚克难求突破，全市经济实现平稳较快发展，社会保持和谐稳定。全年完成地区生产总值1596.2亿元，比2016年（下同）增长9.1%；规模以上工业增加值增长10.9%；全社会固定资产投资2042.1亿元，增长18%；社会消费品零售总额722.1亿元，增长13.3%；地方一般公共预算收入146亿元，增长11.3%；城镇居民人均可支配收入31449元，增长8.6%；农村居民人均可支配收入13670元，增长9.8%，主要经济指标增速位居全省前列。中国（四川）自由贸易试验区川南临港片区揭牌运行，全国文明城市创建一举成功。市八届人大一次会议确定的主要目标任务较好完成，向全市人民承诺的民生实事全部兑现。

一、项目建设和投资拉动势头强劲。认真贯彻“项目投资攻坚年”部署，实施重点项目609个，完成投资1724亿元，占年度投资计划的111.4%。华为四川大数据中心、中航EPS转向系统、合盛硅业泸州基地等产业项目建成投产，沱江二桥加宽改造、沱江六桥等城建项目建成投用，云龙机场、川南城际铁路自贡至泸州段、G8515泸渝高速公路等交通项目加快推进，古蔺观文水库、泸县三星桥水库等水利项目相继建成，市人民医院新院等民生项目竣工投用。强化要素保障，出台“零地”技改政策，全年保障用地3.8万亩，工业用电量43.6亿千瓦时，工业用气量16.6亿立方米，全市金融机构对重点项目贷款余额303.6亿元，较年初增加75.8亿元。实现民间投资1130.5亿元、增长7.5%。全社会固定资产投资增速连续三年居全省第一。

二、产业转型升级取得重大突破。深入推进供给侧结构性改革，大力发展现代制造业，智能终端产业从无到有、初具规模，落户项目93个，总投资380亿元，32个项目建成投产，投产后可实现年销售收入520亿元， “泸州造”智能手机成功走出国门。航空航天、装备制造、现代医药、信息技术、新能源新材料等新兴产业加快培育，航天科工预冷动力技术中心成功签约，泸州金能移动能源项目（一期）开工建设，步长生物制药基地、聚碳酸酯（一期）加快推进，新能源电动专用车正式量产。巩固提升白酒、机械、化工、能源等传统优势产业，泸州老窖酿酒技改工程、郎酒天宝峰陶坛酒库等项目加快建设，组建川酒集团带动中小酒企抱团发展，“四大产业”增加值增长12.9%。加快发展商贸、物流、金融等现代服务业，服务业实现增加值562.4亿元、增长9.5%，步步高综合体、泸州电商产业园、海吉星物流园（一期）建成运营，万达广场加快推进。兴泸水务在香港联交所、华西证券在深圳证交所成功上市，新入驻金融机构7家。旅游业实现总收入441.9亿元、增长33.4%。商品房销售额350.3亿元、增长40.3%。深入推进农业供给侧结构性改革，加快国家现代农业示范区建设，集中打造江阳区董允坝、泸县谭坝等产业融合示范园15个，新发展市级以上重点龙头企业25家，粮食总产量206.1万吨，“八大特色农业产业”稳步发展。加快园区载体建设，全市园区新建标准化厂房155万平方米，实现主营业务收入2248亿元、增长18.3%。“泸州制造”“泸州服务”“泸州品牌”正为全市加快发展注入强劲动力。

三、区域中心城市建设迈出坚实步伐。深入实施国家新型城镇化综合试点，加快推进“两江新城”建设，大力改造提升老城区，中心城区建成区面积154平方公里、人口152万人，城镇化率达49%。大力提升城市功能品质，海绵城市建设试点扎实推进，城市道路“白加黑”升级改造、城区景观照明（一期）工程全面完成，重要节点立交改造项目竣工投用，长江二桥、二环路等项目快速实施，鸭儿凼污水处理厂提标扩容工程建成运行，沱江新城南北滨公园主体完工。施行城市管理领域3部地方性法规，推进城市管理综合执法体制改革，城市管理和公共服务水平不断提高。加快县城和小城镇建设，泸县撤县设区进展顺利，合江、叙永、古蔺城乡面貌显著改善，大渡口镇、江门镇、白节镇列入第一批省级特色小镇。全力创建全国文明城市，深入实施“六大工程”，全市人民参与创建的磅礴力量不断汇聚，实现“三年创建，一举成功”。

四、自贸试验区川南临港片区建设开局良好。突出制度创新，复制推广改革经验158项，形成制度创新成果90项，14项制度全国首创，获批启运港退税政策试点，建成行政审批、国际贸易等 “单一窗口”，进出口通关时间分别降低56%、49%。突出产业聚集，航运物流、港口贸易、智能终端等产业加快发展，新注册企业3041户，实现进出口贸易总额20.6亿美元、增长5.8倍。突出服务川南，实施口岸通关“零费用”、保B仓库“零租金”，推动自贸红利区域共享。突出协同开放，与成都片区建立物流一体化合作机制，开通“泸蓉欧”快铁，推出泸州至上海“天天直航快班”，与广东南沙、天津滨海等17个地区建立“政务服务异地直通互办”网络。川南临港片区为泸州发展增添新动能。

五、改革开放和创新发展成效显著。深化重点领域改革。30项国省改革试点有序推进，率先在全省开展“多证合一”改革，农村土地制度改革三项试点走在全国前列。“放管服”改革深入推进，新增各类市场主体4.2万户、增长20.7%，县域经济持续壮大。加快国有投融资平台公司市场化转型，泸天化改革脱困稳步推进。营改增试点全面推开，为企业减负超过10亿元。全方位扩大开放，引进世界500强、中国500强、行业百强企业12家，“酒博会”“商博会”“农博会”影响力持续扩大。创新创业蓬勃发展，推出“酒城人才新政”，设立每年5000万元的泸州市人才发展基金，新建创新型科研机构10家、产业技术联盟5家，实现高新技术产值315亿元、增长48.7%，西南医科大学科研项目荣获国家科技进步二等奖。

六、脱贫攻坚取得阶段性成效。全年实现8.5万人脱贫、149个贫困村退出，合江县脱贫“摘帽”。创新易地扶贫搬迁“五统一”模式，累计完成搬迁16281户66502人，基本实现“五年任务、两年完成”，叙永县江门镇集中安置点列入国家“砥砺奋进的五年”大型成就展。突出产业扶贫，在全省率先将贫困村产业扶持基金提高到50万元/村，组织3家市属国企打造特色产业示范片，发挥温氏、巨星等农业龙头企业带动作用，贫困地区造血功能持续增强。全域推进“一改三化”，预脱贫村集体经济收入全部达标。基础设施明显改善，建成农村扶贫公路2148.4公里、渡改桥8座、饮水工程1569处，贫困村电网改造升级全面完成。健康扶贫“四个百分之百”全面实施。建成省级“四好村”75个。援藏援彝工作扎实推进。扶贫机制不断健全、力量不断整合，专项扶贫、行业扶贫、社会扶贫“三位一体”的大扶贫格局初步形成。

七、民生和社会事业全面进步。十项民生工程和20件民生实事全面完成，民生支出占一般公共预算支出比重达70.1%。城镇新增就业4.33万人，失业人员再就业1.17万人，新增机构养老床位2500张。危旧房棚户区改造开工10300户。各级各类教育协调发展，新建公办中小学校19所、幼儿园8所、教师周转宿舍1110套，“全面改薄”累计竣工校舍98.1万平方米，免费定向培养村小教师229名，纳溪义务教育均衡发展通过国家评估认定。新建四川城建职业技术学院、四川海事交通职业学院列入《四川省高等学校“十三五”设置规划》。全市中小学幼儿园 “三防”标准化建设基本完成。国家文化消费试点工作扎实推进，“宽带乡村”工程全面实施。深入开展全民预防保健，免费体检178万人，新改建中心村卫生室232个，远程诊疗工程实现五级全覆盖，市人民医院创“三甲”医院通过验收，国家健康城市试点取得阶段性成效。全面从严落实安全生产责任，安全生产形势持续向好。国家食品安全示范城市创建有序开展。依法治市扎实推进，“七五”普法深入开展。实施基层治理专项行动，“一村一辅警”“雪亮工程” 实现全覆盖，社会治安立体化防控体系更加完善。

八、生态环境质量显著提升。大力整治环境突出问题，中央环保督察组交办的227件信访件、省政府交办的188件信访件全部办结，集中式饮用水水源地保护、城市黑臭水体等一批群众关注的问题得到有效解决。坚决打好污染防治“三大战役”，全市地表水国控考核断面水质优良比例85.7%，主城区环境空气优良天数273天、比上年增加40天，工矿废弃地土壤污染治理有序推进。全面落实“河长制”，设立河（段）长5438名，实施12座污水处理厂扩容提标改造，新建截污干管37公里，拆除取缔非法码头54座、规范提升10座，长江、沱江泸州段水质稳定向好。着力推进生态优先绿色发展，制定《绿色发展规划》，全面完成总量减排，综合治理水土流失面积199.8平方公里，森林覆盖率达50.4%，顺利通过国家森林城市复检。

国防动员、人民防空、民兵预备役建设不断加强；青年、妇女儿童、老龄、残疾人事业取得新进步，民族、宗教、外事、侨务、对台、统计、消防、保密、气象、双拥、档案、地方志等工作取得新成效。

过去一年，我们扎实推进法治政府、效能政府、廉洁政府建设，深入推进依法行政，严格执行市人大及其常委会决议决定，自觉接受市政协民主监督，全力做好人大代表建议和政协委员提案办理，办结建议、提案560件，法定时限办复率100%。深入开展“两学一做”学习教育，坚持不懈纠正“四风”，加大行政监察和审计监督，严肃查处政府系统违纪案件，坚决整治损害群众利益的不正之风。

各位代表，2017年全市经济社会发展取得的成就，是省委、省政府和市委正确领导的结果，是市人大、市政协监督支持的结果，是全市上下真抓实干、奋勇拼搏的结果。在此，我代表市人民政府，向全市人民致以崇高的敬意！向人大代表、政协委员，向各民主党派、工商联、各人民团体，向驻泸部队官兵和社会各界人士，表示衷心的感谢！

在肯定成绩的同时，我们也清醒地看到，发展中还存在一些困难和问题：一是传统产业还需进一步转型升级，新兴产业对经济增长的贡献仍然不足，产业投资规模还不够大，民间投资活力还不够强。二是脱贫攻坚任务仍然比较艰巨，产业扶贫带动作用还需进一步加强，农村基础条件有待进一步改善。三是社会治理任务仍然比较艰巨，安全生产、社会治安等领域仍然潜在风险隐患，社会治理的基层基础还需进一步夯实。针对这些问题，我们将在今后工作中采取有力措施，切实加以解决。

2018年主要目标任务

今年是全面贯彻落实党的十九大精神的开局之年，是改革开放40周年。做好今年的政府工作，必须以习近平新时代中国特色社会主义思想为指导，深入贯彻党的十九大精神，牢固树立和贯彻新发展理念，按照省委十一届二次全会、市委八届四次全会决策部署，牢牢把握“稳中求进、进中求快、快中求好”的工作基调，聚焦“项目攻坚、创新发展”工作主题，强力推进“六个突破”，着力“三抓三主动”，统筹抓好稳增长、促改革、调结构、惠民生、防风险各项工作，提高发展质量和效益，坚决打好防范化解重大风险、精准脱贫、污染防治三大攻坚战，加快决胜全面小康、建设现代化区域中心。

经济社会发展的主要预期目标是：地区生产总值比2017年（下同）增长9%，规模以上工业增加值增长10.7%，全社会固定资产投资增长15%，社会消费品零售总额增长12%，地方一般公共预算收入增长9%，城镇居民人均可支配收入增长8.2%，农村居民人均可支配收入增长9.5%。重点做好以下工作：

一、强化项目投资攻坚，全力以赴稳定经济增长

加大项目投资力度。积极谋划和推进一批大项目、好项目，持续发挥项目对稳增长的关键作用。实施5000万元以上重大项目995个，年度完成投资1543.9亿元。确保170个重大项目竣工投用，年度完成投资242.9亿元；加快推进249个重大项目建设，年度完成投资709.5亿元；开工建设368个重大项目，年度完成投资591.5亿元。全社会固定资产投资继续走在全省前列。

突出重点领域投资。狠抓产业投资，完成重大产业项目投资860亿元以上，重点推进206个重大工业项目，年度完成投资321.7亿元。狠抓基础设施建设投资，年度完成投资435.2亿元。坚持交通先行，加快建设全国性综合交通枢纽，确保云龙机场7月建成投运；突出“三路一总站”建设，快速推进川南城际铁路自贡至泸州段建设，力争渝昆高铁、城北高铁枢纽站、泸永高速公路开工建设；启动泸遵高铁前期工作，确保G8515泸渝高速公路、叙威高速公路顺利推进。狠抓民生社会事业、生态环保、精准脱贫等领域投资，为可持续发展奠定坚实基础。

强化项目要素保障。统筹资金、土地等要素，推动资源向优质项目集聚。深入实施“一企一策”，扩大直购电、直供气、富余电量消纳范围，全年减少企业用能成本8亿元以上。加大金融对重点项目支持力度，促进重点企业直接融资；推动银企项目对接常态化，项目贷款增长17%以上。强化国有资本与社会资本合作，激活民间投资活力。

二、推进产业转型升级，加快构建现代产业体系

加快发展先进制造业。聚焦航空航天、智能终端、高端装备制造、现代医药、大数据等新兴产业，加快形成发展新支撑。大力发展航空航天产业，加快航空航天产业园（一期）建设，推进北航泸州研究院、航天科工等航空发动机项目尽快落地，力争沈阳旋飞科技无人机项目开工建设。大力发展智能终端产业，新引进一批手机整机和核心配套企业，新建标准化厂房160万平方米、生活配套设施55万平方米，确保67个项目投产达效，加快打造“千亿产业”。全力促成与德人工智能科技园、中科智富光电项目落地，加快建设朗润飞依诺医疗器械产业化基地、西部绿色智能住宅产业园等项目，确保泸州金能移动能源项目（一期）、江麓容大无级自动变速器研发生产基地（一期）等项目建成投产。加快发展大数据产业，重点培育酒业、物流、医疗健康等大数据，积极争创国家大数据产业集聚区。

优化提升传统优势产业。深化供给侧结构性改革，支持传统企业工艺更新、装备换代、产品升级。做强做优白酒产业，力争全市酒业主营业务收入突破900亿元、增长13%。发挥泸州老窖、郎酒龙头带动作用，加快推进泸州老窖酿酒技改工程、郎酒技改工程建设；川酒集团实现销售收入50亿元、利税5亿元，带动全市中小酒企加快发展，着力培育泸酒“第三极”。转型做精化工产业，加快鼎科纳米脱酸液研发生产基地建设，完成中海沥青与中海金象混改重组，启动100万吨沥青加工技改项目。优化提升能源产业，稳步推进电力体制改革。加快振兴机械产业，启动长江机械扩能技改，推进大型农机高端液压元件、环保设备、智能立体车库研发推广，促进机械产业智能化、服务化升级。

培育发展现代服务业。推动服务业提质增效，加快打造区域商贸、物流、金融、会展中心。大力发展现代商贸业，启动回龙湾商圈改造升级，推进西南商贸城（三期）、华升宝龙广场建设，确保万达广场建成投用，提升城市商圈品质。大力发展现代物流业，推进全国首批多式联运示范城市建设，启动石龙岩现代商贸物流园项目，加快和润粮油物流基地建设。大力发展全域旅游，重点推进天下川江文化公园、福宝国际旅游度假区、方山文旅小镇、玉蟾山国际温泉度假区（二期）等项目建设，旅游总收入突破490亿元。大力发展现代金融业，新引进3家以上金融机构，银行业金融机构各项贷款余额增长12%，推动科瑞德、天植中药上市挂牌。促进房地产和建筑业平稳健康发展。持续提升“春酒、秋商、冬农博”展会影响力。

推进园区提档升级。加快园区生产、生活基础设施建设，全市产业园区主营业务收入突破2600亿元、增长16%。高新区完成投资65.8亿元，加快国家创新型特色园区建设。长开区完成投资59.7亿元，加快推进外向型经济发展。酒业园区完成投资56.5亿元，开工建设环球佳酿等项目，加快推进产城融合、三产互动。航空航天产业园区完成投资41亿元，加快打造泸州航空航天科创新城。

三、深化农业供给侧结构性改革，大力实施乡村振兴战略

大力发展现代农业。坚持质量兴农、绿色兴农，深入实施“十大行动”，建好基地、育好龙头、搞好加工、创好品牌。重点打造15个现代农业产业融合示范园区，推进温氏生猪、正大肉鸡、古叙肉牛等项目建设。大力培育和引进现代农业经营主体，新增市级以上重点龙头企业22家、家庭农场示范场30个。强化农产品精深加工，优化物流运输等配套服务。积极实施农产品商标战略，加快培育一批知名品牌，新打造省级以上农产品品牌5个。抓好高效节水灌溉、骨干水源工程建设和病险水库整治，加强高标准农田建设，稳定粮食生产，确保粮食安全。推进“互联网+现代农业”，扶持发展农村电商、乡村旅游等新产业新业态，推动农村一二三产业融合发展。

加快建设美丽乡村。积极打造乡村田园综合体，强化基础设施、公共服务、环境风貌建设，持续改善农村生产生活条件。新改建通村公路1000公里，确保纳溪区、泸县全部建制村通公交，合江县、叙永县、古蔺县全部建制村通客运。加强农村教育、文化、医疗等基本公共服务建设，建成一批文化设施、体育场所。开展农村人居环境整治，着力补齐农村人居环境短板。健全完善自治、法治、德治相结合的乡村治理体系，提升乡村治理能力。积极创建省级“四好村”，建设幸福美丽新村300个。

全力促进农民增收。深化农业农村改革，完善助农增收制度机制，深入推进“一改三化”，发展壮大村集体经济。健全“基地+农户”“企业+合作社+农户”等利益联结机制，新发展农民合作社示范社20家，促进小农户和现代农业有机衔接。创新乡村人才引进培育使用机制，支持农民工返乡创业，探索“高校+新型职业农民”模式，扎实做好实用技术、职业技能和创业培训，让更多的群众懂技术、会经营、能赚钱。

四、统筹推进新型城镇化，加快建设全国美丽城市

优化城市空间布局。围绕建设“美丽泸州”，完成新一轮城市总体规划修编，促进城市空间、产业布局、功能配套协调发展。突出“双两百”区域中心城市建设，积极推进泸县撤县设区，启动泸川新区、航空航天产业园区等控制性详细规划。开展省级 “城市双修”试点，加强城市设计，优化城市沿江空间布局和公共开放空间。大力发展县域经济，培育发展30个市级特色小（城）镇，加快形成县区追赶跨越、争先进位新格局。积极构建现代化城市综合交通体系，推进中心城区、县城、小城镇互联互通、协调联动发展，加快打造“1小时城市经济圈”。

增强城市承载能力。加快“两江新城”建设，深入实施二环路、第二医教园区、沱江新城整体城镇化等项目，快速推进长江二桥、长江六桥、沱江五桥等跨江通道建设。着力完善城市功能，加快地下综合管廊、海绵城市建设，开工建设城北、城西垃圾压缩中转站，快速推进二道溪污水处理厂、金鸡湾天然气储配站等项目，确保城西客运站建成投用。大力提升城市品质，有序实施江阳蓝田片区、龙马潭小市片区、纳溪永宁片区等老城有机更新，加快推进滨江路延伸打造、城市照明景观提升（二期）等工程，建成沱江新城南北滨路及南北滨公园。中心城区新增面积15平方公里。开展“宜居县城建设行动”，新增县城建成区面积4平方公里。

提升城市治理水平。巩固全国文明城市创建成果，启动国家生态园林城市创建，推进智慧城市、国家信用体系建设示范城市建设。深化城市管理综合执法体制改革，健全城市治理体系，促进城市管理精细化、规范化、智能化。加快城乡环卫一体化建设，建成古叙生活垃圾焚烧发电厂，推进主城区生活垃圾分类收集、集中处置和有机废物协同处理。深化“厕所革命”，开展“靓城行动”，大力整治违法建设、占道经营、乱停乱放等突出问题，净化城市生活环境，完成国家卫生城市第七次复审。

五、奋力推进自贸试验区川南临港片区建设，打造内陆开放型经济新高地

推进制度创新。全面落实试验任务，探索建设内陆自由贸易港。积极争取国家部委、省级部门赋权，推进外商投资管理、“证照分离”等改革，促进投资便利化。完善国际贸易“单一窗口”，探索多式联运“一票制”，促进贸易便利化。推进跨境融资、航运金融等领域改革，积极发展金融新业态，促进金融国际化。实施“审管分离”“管执分离”，完善事中事后综合监管平台，促进监管法治化。加强海关、海事、检验检疫等领域制度创新，培育竞争新优势。

积极服务川南。全力争创国家开放口岸、综合保税区、跨境电商综合试验区，建成泸州港进口肉类指定口岸，着力完善功能平台。继续推进“自贸红利•区域共享”，在政务服务互办、口岸监管互认、物流通道共建等领域深化合作，服务川南经济区一体发展。启动泸州港第二作业区建设，加快自贡至泸州港大件路等项目建设，推进区域互联互通。

深化协同开放。加强与成都天府新区片区、青白江铁路港片区协作，推进物流一体化发展，稳定运行“泸蓉欧”快铁。加强与周边地区协作，促进港口物流互通、口岸通关互认，增强服务成都经济区、攀西经济区和滇东黔北能力。持续深化与沿海沿边沿江口岸协作，推进长江航道泸州段“Ⅲ升Ⅱ”工作。深度融入“一带一路”建设和长江经济带发展，拓展与沿线地区、物流节点城市合作。

加快产业集聚。突出航空航天、智能终端、港口贸易、航运物流、融资租赁等重点产业，针对长三角、珠三角等重点区域，实施精准招商。突出水港特色，大力发展大宗商品交易、进口商品分拨、出口商品集散、进出口加工等开放型经济业态，培育发展总部经济、会展经济、保税贸易、跨境电商、航运金融，促进“泸州造”特色产品远销海外，推动自贸试验区引领泸州走出国门、走向世界。

六、全面深化改革创新，持续释放发展动力活力

深化重点领域改革。深入实施国、省改革试点，大力推进国家农村土地制度改革三项试点、国家知识产权试点城市、企业投资项目承诺制试点等改革。积极开展全面创新改革，落实涉及泸州的8张清单。突出重点领域改革，深化国资国企改革，开展国有资本授权经营试点，推进泸天化改革脱困，确保扭亏为盈；深化财税体制改革，加快建立现代财政制度，加强政府债务管理，推进国有投融资平台公司市场化转型；深化地方金融改革，坚决有效防控金融风险。

大力推进创新创业。实施创新驱动发展，加快创新型城市建设，新建创新型科研机构10家、产业技术联盟5家，新培育省级以上科技企业孵化载体8家以上。大力实施高新技术企业倍增计划，新增国家高新技术企业12家，高新技术产值达360亿元。深入实施“酒城人才新政”，举办“中国酒城泸州人才发展大会”，加快人才集聚。扎实开展“酒城创业行动”，持续推动大众创业万众创新。大力弘扬企业家精神，营造企业家健康成长良好环境，鼓励更多市场主体发展实体经济。

加快建设“政务服务示范城市”。深入实施“放管服”改革，以“五通”工程为主抓手，推进“互联网+政务服务”应用。清理整合全市政务服务热线，实现“一号通”；推进全市医疗、保险等便民服务功能集成，实现“一卡通”；推进政务服务数据全面共享，实现 “一网通”；推进各级政务服务综合窗口设置，实现“一窗通”；推进办事“零跑路”和“最多跑一次”，实现“一次通”。着力打造流程最简、效率最高、服务最好的政务服务环境。

七、聚焦精准扶贫精准脱贫，坚决打赢脱贫攻坚战

精准实施产业扶贫。坚持把产业扶贫作为脱贫攻坚的主攻方向，精准制定产业扶贫举措。扶持发展特色产业，重点打造一批“贫困村+非贫困村”跨区域脱贫产业园，加快建设古蔺金鱼溪、叙永黄金榜、合江白村三个农旅融合扶贫示范片，扶持生产和就业发展2.69万人。积极推进资产收益扶贫、飞地扶贫、扶贫车间等新模式，探索资金资产资源“三权”分离新路子。用好用活产业扶持基金，推广“扶贫再贷款+扶贫小额信贷”模式，提升金融信贷对产业扶贫的支撑。

强化基础设施建设。加大涉农资金整合力度，切实改善贫困地区交通、水利、电力、通信等基础设施条件。全面完成“十三五”期间易地扶贫搬迁住房建设任务。推进农村扶贫公路项目，加快建设赤水河环线扶贫公路，解决2.6万名建档立卡贫困人口饮水困难问题，全面完成贫困村农网改造，实现“组组通”宽带。确保所有预脱贫村通硬化路，有文化室、卫生室、通信网络。

突出抓好健康扶贫。强化建档立卡贫困人口健康体检，实现精准到户、精准到人、精准到病。聚焦“基本医疗有保障”，扎实开展健康扶贫“五大行动”，全面落实“四个百分之百”，建好、用好医疗扶贫救助基金，切实减轻困难群众就医负担，防止因病致贫、因病返贫发生。

持续巩固脱贫成果。壮大“四项基金”规模，提高资金投入精准性。全面落实教育扶贫政策，确保贫困家庭学生不因家庭经济困难失学。巩固低保政策，确保困难群众生活得到保障。坚持扶贫与扶志、扶智相结合，办好农民夜校，增强贫困群众自我发展能力。扎实做好定点扶贫和驻村帮扶，提升贫困村“五个一”、非贫困村“三个一”帮扶实效。强化“回头看”“回头帮”，巩固脱贫成效。全力确保5.87万名农村贫困人口脱贫，86个贫困村退出。

八、加强生态文明建设，推进生态优先绿色发展

坚决打好污染防治“三大战役”。大力实施大气环境质量提升工程，深入治理扬尘、秸秆焚烧、机动车和工业废气等污染，促进全市空气质量持续好转。大力实施水污染防治“四大行动”，强化城镇生活污染治理和农业农村污染防治，加快城市备用水源地建设，完成乡镇集中式饮用水水源地污染整治和规范化建设。开展土壤污染状况详查，大力实施矿山地质环境修复治理。以“零容忍”态度坚决打击生态环境违法行为，让泸州天更蓝、地更绿、水更清。

深入推进“河长制”。严格实施“一河一策”，全面落实河长制“六大任务”。开展河道管理范围划定，进一步明晰管理边界。建成以河长制管理模式为核心的河湖管理信息平台，完善水质、水情监测预报体系。全面启动“湖长制”，探索建立河道警长制，严厉打击涉水违法犯罪行为。加快沱江流域泸州段污染整治重点项目建设，确保沱江水质主要指标达到III类标准，长江出川断面水质稳定保持在III类标准以上。

着力加强生态建设。开展生态保护红线划定，严格保护耕地。推进新一轮“绿化泸州行动”，持续加大天然林资源保护，完成营造林37万亩，全市森林覆盖率达50.6%。综合治理水土流失面积160平方公里，治理岩溶石漠化面积76平方公里。加强长江上游珍稀特有鱼类等自然保护区监管。强化生态环境监测预警、联防联控、应急处置，严格执行生态环境损害责任终身追究制。

加快推进绿色发展。优化沿江产业布局，推动集中集约集群发展。加快节能环保装备研发推广，推进绿色建筑产业集聚示范城建设。引导鼓励企业提标升级，实施传统制造业绿色改造，积极化解过剩产能，全面完成节能减排目标任务。倡导绿色生产生活方式，推动绿色低碳循环发展。

九、大力发展社会事业，千方百计改善民生

深入实施民生工程。持续加大民生投入，确保民生投入占一般公共预算支出的比重保持在70%以上。扎实推进十项民生工程，办好20件民生实事。加快完善各项就业政策，新增城镇就业3.6万人，确保城镇登记失业率控制在4%以内。深入开展全民参保登记，完善城乡居民大病医疗保险办法。实施农村危房改造1.2万户，城镇危旧房棚户区改造开工9216户。开工建设“渡改桥”3座，建成5座。

大力发展社会事业。深化教育体制机制改革，推进学前教育普惠健康发展、县域内城乡义务教育一体化发展，实施高中阶段教育普及攻坚计划，稳步提升教育发展质量。深入实施“全面改薄”，新建公办中小学校5所、公办幼儿园24所。大力发展职业教育，支持泸州职业技术学院建设四川省优质高等职业院校。培养高素质教师队伍，加强乡村教师队伍建设。强化校园及周边环境治理，打造平安校园。深化文化消费试点，推进高清泸州•智慧广电、城市影院、城市博物馆群、科技馆群建设，打造15分钟公共文体服务圈。深化基层文化体育事业发展，新建村、社区体育健身工程160个，开展全民健身、文化惠民等活动，不断满足群众精神文化生活需求。

加快建设健康泸州。进一步完善全民预防保健服务体系，探索实施主动健康工程，加快创建全国健康城市示范市。深化医药卫生体制改革，扶持和发展中医药事业，加快推进西南医疗康健中心（二期）、市中心血站迁建等项目建设，建立优质高效的医疗卫生服务体系。深化养老服务综合改革，推进医养融合发展。加强食品药品质量安全监管，加大对食品药品虚假宣传等违法行为打击力度，积极创建国家食品安全示范城市。

扎实推进基层治理。牢固树立底线思维和红线意识，坚决守住社会稳定、安全生产、食品药品安全“三条底线”。严格落实属地治理责任、部门（单位）包保责任，切实维护经济建设、信访、金融、文教卫生等领域良好秩序。健全落实安全生产责任体系，加强风险管理和隐患排查治理，强化煤矿及非煤矿山、道路交通、危化品、建筑施工、消防等重点行业安全监管，坚决遏制重特大事故发生。加强职业病危害防治，新增城市应急避难场所6个，做好水文、气象监测预报，强化防汛减灾和地灾防治。深化依法治理，推进“平安泸州”建设，健全立体化社会治安防控体系，严厉打击黑恶势力，开展危爆物品、寄递物流、禁毒防邪等专项整治。深入推进“雪亮工程”、四级综治中心规范化建设，实施网格化服务管理，完善矛盾纠纷多元化解机制，确保社会和谐稳定。

积极支持国防和军队改革，加强国防动员和后备力量建设，加快泸州国防教育基地建设，推进新一届省级双拥模范城（区、县）创建。推动援藏援彝工作取得新成效。积极发展妇女儿童、老龄和残疾人事业，切实做好人民防空、民族宗教、外事、侨务、对台、保密、统计、档案、地方志等工作。

十、加强政府自身建设，着力建设人民满意的政府

深化政府系统政治建设。坚持以习近平新时代中国特色社会主义思想统领政府各项工作，全面加强政府系统政治建设。牢固树立“四个意识”、坚定“四个自信”，认真开展“不忘初心、牢记使命”主题教育，推动政府系统更加自觉地落实党中央、国务院、省委、省政府和市委决策部署，提升政府各项工作水平。

深化法治政府建设。严格执行重大行政决策程序规定，进一步深化行政执法“三项制度”，加强行政人员法制教育，切实提高依法行政能力和水平。突出城乡规划、白酒历史文化保护等重点领域，推进政府立法。深化“两法衔接”，扎实推进依法行政。深化政务公开，健全政府新闻发布机制，完成全国基层政务公开标准化规范化试点。认真执行市人大及其常委会的决议、决定，自觉接受市人大、市政协监督，切实提高人大代表建议、政协委员提案办理质量和效率。

深化效能政府建设。坚持从严从实，持之以恒抓好效能建设，进一步提升政府系统执行力、公信力。加强公务员队伍能力素质建设，注重培养专业能力和专业精神。大力践行“一线工作法”，精准破解发展中的困难问题。强化政务督查、绩效管理和履职问责，完善正向激励和容错纠错机制，坚决整治不作为、乱作为、慢作为，推动各项工作干在实处、走在前列。

深化廉洁政府建设。从严落实廉政建设责任制，有力推进政府系统反腐倡廉工作。坚决贯彻落实中央八项规定精神和省委省政府十项规定精神实施细则，严防“四风”反弹。强化审计监督和社会监督，加强重点领域、关键环节和重要岗位权力运行的监督管理，着力营造风清气正的良好氛围。

各位代表！进入新时代、开启新征程，我们深感责任重大、使命光荣。让我们更加紧密地团结在以习近平同志为核心的党中央周围，在省委、省政府和市委的坚强领导下，紧紧依靠全市人民，不忘初心、牢记使命，开拓创新、拼搏奋进，为泸州决胜全面小康、建设现代化区域中心而努力奋斗！
